# Channel and Body-Diode Conduction Characteristics in 4H-SiC MOSFETs Under Third-Quadrant Switching Conditions

**DOI:** 10.3390/mi17050526

**Published:** 2026-04-25

**Authors:** Xiaobing Huang, Yihui Song, Chiyu Zhong, Zhigang Wang

**Affiliations:** 1 School of Information Science and Technology, Southwest Jiaotong University (SWJTU), Chengdu 611756, China; 2 School of Integrated Circuits Science and Engineering, Southwest Jiaotong University (SWJTU), Chengdu 611756, China; zhong2025@my.swjtu.edu.cn (C.Z.); zhigangwang@swjtu.edu.cn (Z.W.)

**Keywords:** reverse conduction, reverse recovery characteristics, SiC MOSFET, third-quadrant operation

## Abstract

The third-quadrant operation of silicon carbide (SiC) MOSFETs is investigated from the perspective of carrier transport, focusing on the interaction between two parallel conduction paths. Through experimental characterization and TCAD simulation, the conduction behavior of the PiN body diode and MOS channel under various gate-source bias conditions is examined. Results reveal that body-effect-induced threshold voltage (*V*th) reduction enables channel conduction even under negative gate bias. Based on this mechanism, a transfer-characteristic-based method is developed to identify gate-voltage boundaries between conduction modes. The impact of negative gate bias on reverse recovery parameters, peak current (*I*_rr_), charge (*Q*_rr_), and time (*t*_rr_), is quantitatively evaluated. At the unit-cell level, current sharing between the two paths is analyzed, clarifying the physical mechanism governing their redistribution.

## 1. Introduction

Silicon carbide (SiC) MOSFETs have been widely used in various applications due to their low conduction loss, high switching speed, and superior high-temperature capability [1,2,3]. In converter operation, these devices conduct current in the third-quadrant during freewheeling intervals, where load current flows from source to drain [4,5]. The intrinsic body diode provides a natural freewheeling path, making third-quadrant behavior essential for loss estimation, reliable circuit design, and potential elimination of external Schottky barrier diodes to reduce cost and improve power density.

It is known that reverse current (*I*_SD_) in SiC MOSFETs flows through two parallel paths: the intrinsic PiN body diode and the MOS channel [6]. These paths exhibit fundamentally different conduction mechanisms. The MOS channel operates as a unipolar device conducting through majority carriers, while the PiN body diode relies on bipolar conduction with minority-carrier injection and storage [7]. Due to this distinction, the voltage drop across the MOS channel can be significantly lower than that of the body diode under appropriate gate bias, enabling the device to operate in a synchronous rectification mode that substantially reduces reverse conduction losses compared to the natural freewheeling mode [8].

The third-quadrant characteristics of SiC MOSFETs have attracted considerable research interest. Some studies identify the two conduction paths and qualitatively describe their current-sharing behavior [9,10,11]. Building on this foundation, other studies reveal that the MOS channel can remain partially active even under zero or moderately negative gate bias due to the body effect [12,13]. This body-effect-induced reduction in the effective threshold voltage (*V*_th,eff_) creates a transitional conduction regime where current gradually transfers from the PiN path to the channel path as the source voltage increases [7]. The influence of channel length, temperature, and device structure (planar and trench) on third-quadrant behavior has also been explored [5,14,15].

This dual-path conduction affects both static characteristics and dynamic performance. Reverse recovery behavior is influenced by gate bias through current sharing: channel participation reduces minority-carrier injection into the drift region, lowering reverse recovery charge (*Q*_rr_) and time (*t*_rr_) [7]. Conversely, when the channel is fully suppressed and all current flows through the PiN path, stronger minority-carrier storage results in more pronounced reverse recovery. Therefore, understanding the quantitative correlation between gate bias, current partitioning, and reverse recovery parameters is particularly important for applications where negative gate bias is used to enhance noise immunity and reduce switching losses, as turn-off voltage directly impacts both static and dynamic third-quadrant behavior.

In this paper, the gate-dependent third-quadrant behavior of SiC MOSFETs is investigated from a physics perspective. Section 2 describes the device structure and the experimental methodology. Section 3 presents experimental and TCAD simulation results, analyzing carrier transport mechanisms, conduction path interaction, and the influence of negative gate bias on reverse recovery dynamics. Section 4 concludes this paper.

## 2. Device Structure and Methodology

**Figure 1:**
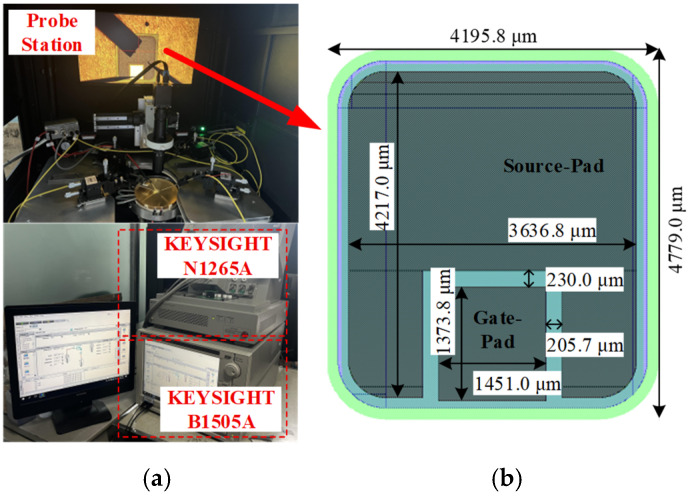
(**a**) Experimental test setup. (**b**) Chip layout of the SiC MOSFET used in the experiment.

The experimental setup and device layout of the 4H-SiC MOSFET are illustrated in Figure 1, and the key unit-cell parameters used in the simulations are summarized in Table 1. Based on these design parameters, the devices are fabricated with the carrier lifetime of the 4H-SiC epitaxial layer precisely calibrated to 1.2 μs, and the interface state density of the SiC/SiO_2_ gate stack is controlled at around 5 × 10^11^ cm^−2^·eV^−1^ through process optimization. In particular, the P-well region is formed by three steps of ion-implantation with doses of 3 × 10^12^, 1 × 10^13^, and 4 × 10^13^ cm^−2^ at energies of 80, 270, and 540 keV, respectively, followed by annealing at 1600 °C. The gate oxide thickness is controlled to 40 nm during fabrication. A self-aligned process is adopted for the fabrication of the SiC MOSFET.

**Figure 2:**
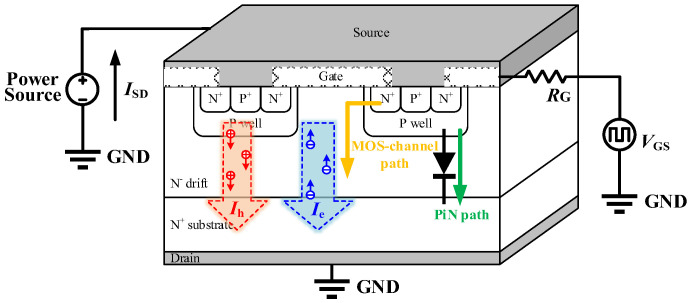
Current conduction paths of the SiC MOSFET in the third quadrant and the corresponding test circuit.

Figure 2 illustrates the structure of a planar SiC MOSFET and the two current paths involved in third-quadrant operation: the intrinsic PiN path and the MOS channel path. The body diode is formed by the P-well, N^−^ drift region, and N^+^ substrate. When the MOS channel is fully turned off, the reverse current (*I*_SD_) flows primarily through the PiN path, resulting in the injection and storage of minority carriers in the N^−^ drift region. During the transition from reverse conduction to forward blocking, a reverse bias is applied across the body diode, and the stored carriers in the N^−^ drift region are rapidly extracted, producing the reverse recovery current (*I*_rr_) peak. This current originates from the dynamic removal of stored charge in the N^−^ drift region. The reverse current *I*_SD_ can therefore be expressed as:

(1) $$I_{\mathrm{SD}}(t)=I_{\mathrm{DC}}+\frac{dQ_{n}(t)}{dt}$$

where *I*_DC_ is the steady-state forward current of the body diode, and *Q*_n_(*t*) is the stored electron charge in the N^−^ drift region. The stored charge *Q*_n_(*t*) can be expressed as:

(2) $$Q_{n}(t)=qA\int_{W_{\mathrm{dep}}(V_{\mathrm{SD}}(t))}^{W}n(x,t)dx$$

where *W*_dep_ is the depletion width under source-to-drain voltage (*V*_SD_), and *W* is the thickness of the N^−^ drift region. The carrier distribution *n*(*x*, *t*) follows an exponential profile:

(3) $$n(x,t)=p_{\mathrm{inj},\mathrm{NQS}}(t)\exp\left(-\frac{x}{L}\right)$$

where *p*_inj,NQS_(*t*) is the injected carrier concentration at the P-well/N^−^ drift junction under non-quasi-static (NQS) conditions, and *L* is the diffusion length related to the carrier lifetime. To account for the delayed release of stored carriers during reverse recovery, the injection level follows:

(4) $$\begin{array}{c} p_{\mathrm{inj},\mathrm{NQS}}(t)=p_{\mathrm{inj},\mathrm{NQS}}(t-\Delta t)+\\ \frac{\Delta t}{\tau_{\mathrm{NQS}}+\Delta t}\times \left(p_{\mathrm{inj},\mathrm{NQS}}\left(V_{\mathrm{SD}}(t)\right)-p_{\mathrm{inj},\mathrm{NQS}}(t-\Delta t)\right) \end{array}$$

where *p*_inj,NQS_(*t* − Δ*t*) is the injected carrier concentration at the previous time step (*t* − Δ*t*). Equation (4) indicates that the carrier release process is limited by the carrier transport time constant *τ*_NQS_.

The two conduction paths act as parallel elements in the unit cell. The total reverse current is the sum of the channel current and the PiN diode current:

(5) $$I_{\mathrm{total}}=I_{\mathrm{ch}}(V_{\mathrm{GS}},V_{\mathrm{DS}})+I_{\mathrm{PiN}}(V_{\mathrm{DS}})$$

The channel current *I*_ch_ in the subthreshold regime follows the exponential dependence:

(6) $$I_{\mathrm{ch}}=I_{\mathrm{ch}0}\exp\left(\frac{V_{\mathrm{GS}}-V_{\mathrm{th}}(V_{\mathrm{SB}})}{nV_{T}}\right)\left[1+\exp\left(-\frac{V_{\mathrm{DS}}}{V_{T}}\right)\right]$$

where *n* is the subthreshold swing coefficient, *V*_T_ is the thermal voltage, and *V*th(*V*_SB_) is given by:

(7) $$V_{\mathrm{th}}(V_{\mathrm{SB}})=V_{\mathrm{th}0}+\gamma \left(\sqrt{2\phi_{F}+V_{\mathrm{SB}}}-\sqrt{2\phi_{F}}\right)$$

The PiN diode current follows the ideal diode equation:

(8) $$I_{\mathrm{PiN}}=I_{\mathrm{SD}}\left[\exp\left(\frac{V_{\mathrm{DS}}}{n_{\mathrm{PiN}}V_{T}}\right)-1\right]$$

The current partitioning factor *α* is defined as the fraction of current flowing through the PiN path:

(9) $$\alpha =\frac{I_{\mathrm{PiN}}(V_{\mathrm{DS}})}{I_{\mathrm{total}}}=\frac{I_{\mathrm{PiN}}(V_{\mathrm{DS}})}{I_{\mathrm{ch}}(V_{\mathrm{GS}},V_{\mathrm{DS}})+I_{\mathrm{PiN}}(V_{\mathrm{DS}})}$$

This formulation captures the continuous transition from pure PiN conduction (*α* = 1 when the channel is fully suppressed) to mixed conduction (0 < *α* < 1 when the channel participates) as *V*_GS_ increases from negative values toward zero. For instance, at *V*_GS_ = −5 V, the MOS channel is completely suppressed, and *I*_SD_ flows almost entirely through the PiN path (*α* = 1). At *V*_GS_ = 0 V, partial channel conduction leads to current sharing between the MOS channel and the PiN path (0 < *α* < 1). The effective carrier injection into the N^−^ drift region can therefore be written as:

(10) $$p_{\mathrm{inj},\mathrm{PiN}}(V_{\mathrm{GS}})=\alpha \cdot p_{\mathrm{inj},\mathrm{eff}}$$

As *V*_GS_ increases from −5 V to 0 V, *α* decreases, reducing the stored charge in the N^−^ drift region and consequently lowering both the *Q*_rr_ and *t*_rr_. Substituting (10) into (2) and (3), the gate-dependent *Q*_rr_ can be expressed as:

(11) $$Q_{\mathrm{rr}}(V_{\mathrm{GS}})=\alpha \cdot Q_{\mathrm{rr},\mathrm{PiN}}$$

where *Q*_rr,PiN_ is the *Q*_rr_ when all current flows through the PiN path (*V*_GS_ = −5 V). This linear scaling relationship provides a quantitative link between the static current partitioning and the dynamic reverse recovery behavior.

This gate-dependent redistribution of current also explains the wide transitional conduction region observed in SiC MOSFETs. When *V*_GS_ exceeds a certain inflection point under negative bias, the *V*_SD_ begins to decrease rapidly as the MOS channel gradually forms. During this transition, *I*_SD_ is progressively transferred from the PiN diode to the MOS channel. To experimentally capture this behavior, a body diode transfer-characteristic measurement is introduced. In this method, a constant reverse current *I*_SD_ pulse is applied while sweeping *V*_GS_ from negative to positive bias, as shown in Figure 2. The simulated and measured results are presented and discussed in Section 3.

## 3. Results and Discussion

### 3.1. Third-Quadrant Characteristics

**Figure 3:**
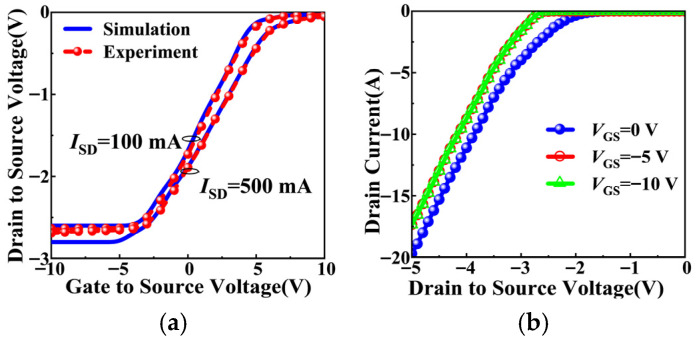
Third-quadrant characteristics of the SiC MOSFETs. (**a**) Measured and simulated results at low (*I*_SD_ = 100 mA) and high (*I*_SD_ = 500 mA) source currents. (**b**) Measured results under different gate-to-source voltages (*V*_GS_).

As shown in Figure 3a, the measured and simulated third-quadrant characteristics are consistent with the three conduction regimes defined by *V*_GS_. At *I*_SD_ = 100 mA, in the diode conduction regime (*V*_GS_ < −5 V), the MOS channel is fully suppressed, and *I*_SD_ flows entirely through the PiN body diode, resulting in a high and nearly constant *V*_SD_. As *V*_GS_ increases into the mixed conduction regime (−5 V < *V*_GS_ < *V*th), the partial channel conducts, and *V*_SD_ begins to decrease as the channel shares part of the reverse current. In the MOS channel conduction regime (*V*_GS_ ≥ *V*th), the channel fully turns on, and *V*_SD_ drops to a low value determined by the channel resistance.

To further verify this behavior, the third-quadrant characteristics are measured under different *V*_GS_ at *I*_SD_ = 100 mA, as shown in Figure 3b. The curves corresponding to *V*_GS_ = −5 V and *V*_GS_ = −10 *V*_GS_ nearly overlap, indicating that the MOS channel is already fully suppressed at *V*_GS_ = −5 V. Under this condition, the *I*_SD_ flows exclusively through the intrinsic PiN body diode, which is consistent with the previous analysis.

**Figure 4:**
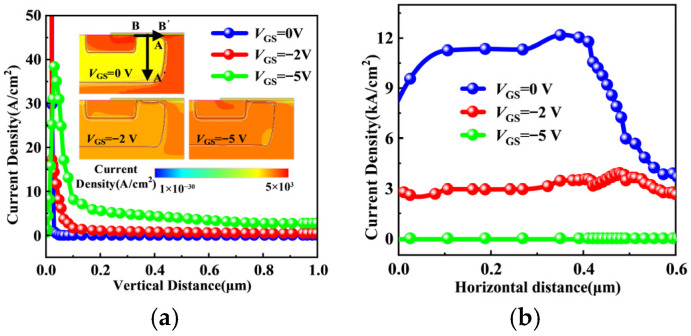
Current density along the (**a**) AA′ line (PiN path), and (**b**) BB′ line (MOS channel path). Insets: The simulated current density distributions.

To clarify internal current distribution, TCAD simulations of third-quadrant operation are performed. The insets of Figure 4a show current density distributions at *I*_SD_ = 100 mA under different gate biases: *V*_GS_ = 0 V, −2 V, and −5 V. Figure 4 presents current density profiles along AA′ (PiN path) and BB′ (MOS channel path) lines marked in the insets. As shown in Figure 4a, the PiN path conducts current under all bias conditions. In contrast, Figure 4b reveals the gate-voltage dependence of MOS channel conduction. At *V*_GS_ = 0 V and −2 V, significant current density along BB′ confirms partial MOS channel conduction. At *V*_GS_ = −5 V, current along BB′ vanishes, indicating complete channel suppression, with *I*_SD_ flowing almost exclusively through the PiN path. This gate-dependent redistribution clearly demonstrates conduction path reconfiguration during third-quadrant operation.

**Figure 5:**
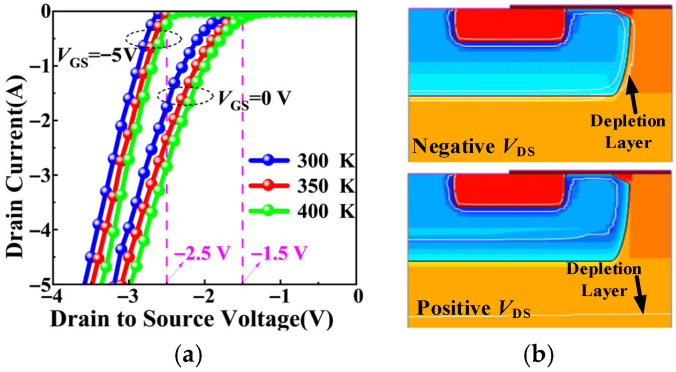
(**a**) Third-quadrant characteristics at different junction temperatures. (**b**) The depletion region of the SiC MOSFET under negative and positive *V*_DS_.

Figure 5a shows the simulated third-quadrant characteristics at different junction temperatures for *V*_GS_ = 0 V and −5 V. For a given temperature, the *V*_GS_ = 0 V curve exhibits a lower turn-on voltage than the *V*_GS_ = −5 V, because partial channel conduction provides an additional parallel path. The steeper slope of the −5 V curve reflects the strong conductivity modulation in the N^−^ drift region under pure PiN conduction, whereas the 0 V curve shows a higher differential resistance due to the absence of such modulation in the channel path. As the temperature increases, both curves shift toward lower *V*_SD_. Although this shift appears similar in the *I*–*V* characteristics, it originates from two distinct physical mechanisms: the negative temperature coefficient of the built-in potential for the −5 V case (pure PiN), and the negative temperature coefficient of the threshold voltage for the 0 V case (mixed conduction).

**Figure 6:**
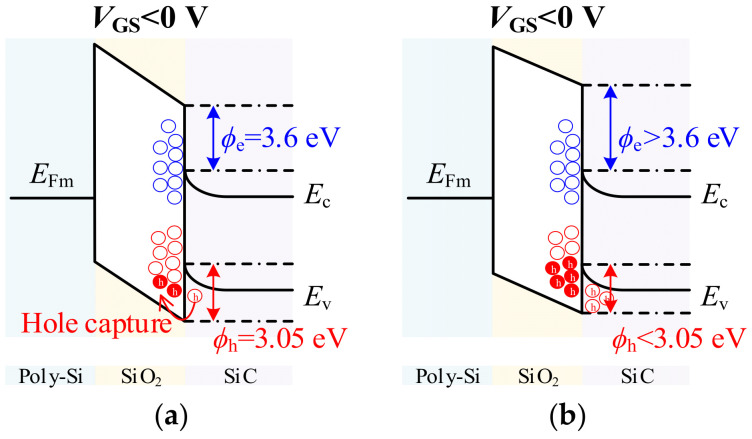
(**a**) Interface trap filling process under negative *V*_GS_: as holes are captured by interface traps, the surface potential gradually shifts, reducing the threshold voltage over time. (**b**) Hole accumulation in the P-well: under sustained negative *V*_GS_, hole concentration in the P-well increases, raising the P-well potential and further modulating the body effect.

Figure 5b compares the simulated electrostatic conditions at *V*_GS_ = 0 V under negative and positive *V*_DS_. When *V*_DS_ is negative (third-quadrant), the depletion region at the P-well/N^−^ drift junction contracts, exposing the channel. The forward-biased junction raises the P-well potential, making *V*_SB_ negative. This negative *V*_SB_ reduces the threshold voltage through the body effect, as described in (5), enabling *I*_SD_ conduction via the MOS channel. This body effect can be further enhanced dynamically. Under continuous negative *V*_GS_, holes accumulate in the P-well, gradually raising its potential over time, as shown in Figure 6b. This transient behavior is described by:

*V*_P_(*t*) = *V*_P,ss_ + (*V*_P,0_ − *V*_P,ss_)exp(−*t*/*τ*_trap_) (12)

where *V*_P0_ is the initial P-well potential, *V*_P,ss_ is the steady-state value, and *τ*_P_ is the hole accumulation time constant. The rising *V*_P_ reduces the effective source-body voltage *V*_SB,eff_ = *V*_S_ − *V*_P(t)_, further lowering *V*th and increasing channel current over time.

Conversely, when *V*_DS_ is positive, the depletion region expands and shields the channel, suppressing conduction. This asymmetric depletion behavior explains why channel conduction occurs during third-quadrant operation, whether from static reverse bias or dynamic gate stress, while remaining blocked in the forward-blocking state. It also clarifies why MOS channel current exists even at *V*_GS_ = 0 V.

### 3.2. Reverse Recovery of SiC MOSFET Body Diode

**Figure 7:**
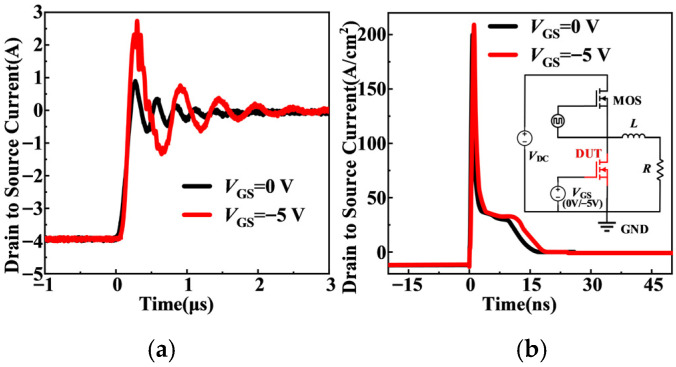
Body diode reverse recovery current: (**a**) experimental, (**b**) simulated results. Inset: the reverse recovery test circuit.

Although the *I*_rr_ of SiC MOSFETs is relatively small, it becomes increasingly important in high switching-frequency applications. Therefore, the influence of negative *V*_GS_ on the reverse recovery characteristics of the body diode is further investigated. The test circuit is shown in the inset of Figure 7b. The *V*_GS_ of the device under test (DUT) is set to *V*_GS_ = 0 V and −5 V, respectively, with *V*_DC_ = 200 V. Initially, the top-side switch turns on to build inductor current. After the current reaches the desired value, the top-side switch turns off. When the high-side switch subsequently turns on, the body diode of the DUT undergoes reverse recovery, generating a reverse current in the opposite direction. The measured and simulated *I*_rr_ result is shown in Figure 7. The extracted *Q*_rr_ and *t*_rr_ values are summarized in Table 2.

As shown in Figure 7, the *I*_rr_ at *V*_GS_ = 0 V is smaller than that at *V*_GS_ = −5 V. This difference originates from the redistribution of the reverse current between the MOS channel path and the PiN path. At *V*_GS_ = 0 V, as analyzed before, a portion of the reverse current flows through the MOS channel while the remainder flows through the PiN path. Since the channel path involves only majority-carrier transport and does not rely on minority-carrier storage, the reverse recovery process mainly consists of rapid majority-carrier extraction, resulting in smaller *Q*_rr_ and shorter *t*_rr_. In contrast, at *V*_GS_ = −5 V, the MOS channel is completely turned off, and the *I*_SD_ flows entirely through the PiN path, relying on minority-carrier injection and storage. Subsequent removal of stored charge leads to larger *Q*_rr_, longer *t*_rr_, and a higher *I*_rr_ peak.

As summarized in Table 2, compared with the case of *V*_GS_ = −5 V, operation at *V*_GS_ = 0 V results in a 25% reduction in *Q*_rr_ and a 12% decrease in *t*_rr_, confirming that channel participation effectively reduces minority-carrier storage and accelerates the reverse recovery process. These results are consistent with the current partition model in (9).

**Figure 8:**
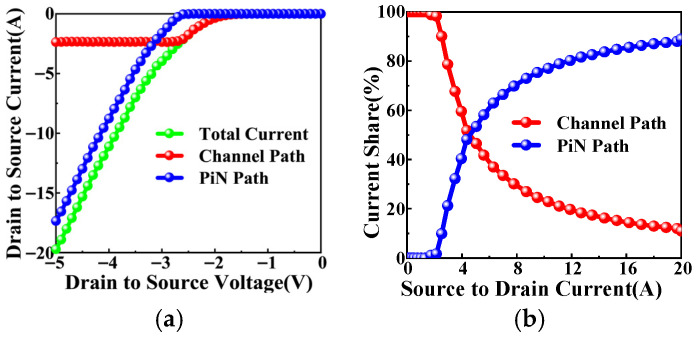
Calculated current sharing between the channel path and PiN path at *V*_GS_ = 0 V: (**a**) absolute values, (**b**) percentages.

As shown in (9), the current partitioning between the two parallel conduction paths is not only gate-dependent but also strongly modulated by *V*_SD_. The current partitioning at *V*_GS_ = 0 V is shown in Figure 8 as a function of *V*_SD_. As *V*_SD_ increases, the channel current initially rises but then saturates. This saturation results from two effects: the rising P-well potential reduces the effective gate overdrive, and the expanding JFET depletion region increases channel resistance. In contrast, the PiN current increases monotonically because the P-well/N^−^ drift junction becomes more forward-biased, enhancing minority-carrier injection and conductivity modulation. Consequently, the PiN path increasingly dominates at higher *V*_SD_, reflecting the competition between the gate-controlled channel and the junction-controlled bipolar path.

### 3.3. Effect of Negative Gate Pulses on Third-Quadrant Reverse Conduction

**Figure 9:**
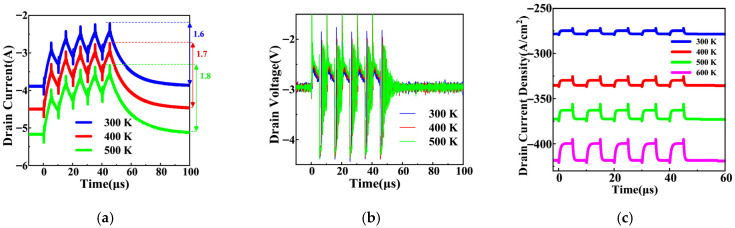
Effect of negative *V*_GS_ on reverse conduction of the SiC MOSFET at different temperatures: (**a**) measured current waveforms, (**b**) measured voltage waveforms, (**c**) simulated current density waveforms.

Based on the test setup in Figure 1, the effect of negative *V*_GS_ on the reverse conduction of the SiC MOSFET is investigated. Figure 9a,b presents the measured results for the SiC MOSFET under third-quadrant reverse conduction at *V*_DS_ = −3 V, with five pulses of *V*_GS_ = −5 V (5 µs width, 50% duty cycle). As the temperature increases, the reverse current variation induced by negative *V*_GS_ pulses significantly increases, and the drain current and drain voltage both exhibit more pronounced changes at higher temperatures. In addition, Figure 9c shows the simulated third-quadrant reverse conduction characteristics of the SiC MOSFET at *V*_DS_ = −3 V, with five pulses of *V*_GS_ = −5 V (5 µs width, 50% duty cycle). The simulated results are consistent with the experimental measurements, showing that the reverse current fluctuation decreases at lower temperatures.

**Figure 10:**
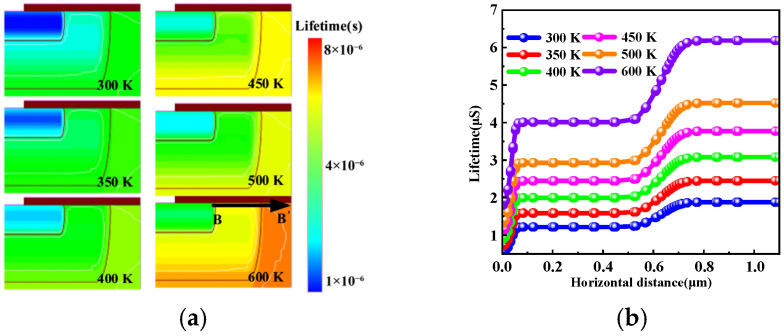
(**a**) Carrier lifetime distribution *V*_GS_ = 0 V. (**b**) Carrier lifetime profile along the BB′ line.

Figure 10a depicts the simulated carrier lifetime distribution at different temperatures with *V*_GS_ = 0 V, and Figure 10b further illustrates the carrier lifetime profile extracted along the BB′ cutline, which corresponds to the MOS channel path marked in Figure 10a. Both figures demonstrate that the carrier lifetime increases with temperature. At the cell level, this phenomenon originates from temperature-induced modulation of parallel conduction mechanisms. Under *V*_GS_ = 0 V, the body effect places the channel in the subthreshold conduction. At elevated temperatures, the extended minority-carrier lifetime and enhanced injection efficiency strengthen carrier storage in the N^−^ drift region, causing the PiN path to carry a larger transient current during the gate pulses. Therefore, higher temperatures significantly enhance the modulation effect of gate pulses on the third-quadrant reverse conduction current, reflecting a pronounced thermal dependence.

**Figure 11:**
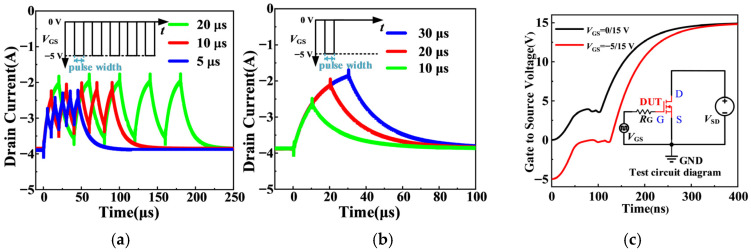
Effect of negative *V*_GS_ with different widths on reverse conduction of the SiC MOSFET: (**a**) experimental current density, (**b**) simulated channel current density under *V*_GS_ of varying widths, (**c**) gate-source voltage waveforms and test circuit diagram of SiC MOSFET under different *V*_GS_.

To further explain the influence of gate bias on the third-quadrant reverse conduction characteristics of SiC MOSFETs, continuous negative gate pulses of −5 V are applied while the drain-source voltage is maintained at a constant *V*_DS_ of −3 V, and the pulse width is varied to change the pulse frequency. The measured results are shown in Figure 11a. The inset shows a schematic diagram of the gate pulse, with a duty cycle of 50%. The results indicate that as the width of the continuous negative gate pulses increases, the steady-state saturation value of the drain reverse conduction current gradually rises, demonstrating a clear pulse-width-dependent behavior. This observation implies that even with a constant gate voltage amplitude, the temporal characteristics of the gate bias can significantly modulate the device’s internal conduction state and, consequently, affect the third-quadrant reverse turn-on current.

Figure 11b summarizes the measured outcomes under a fixed drain bias of −3 V, where negative gate pulses of −5 V with varying pulse widths are imposed. The inset provides a schematic illustration of the adopted gate pulse configuration. With the pulse amplitude kept constant, the reverse conduction current amplitude gradually rises as the negative gate pulse width increases, revealing that the influence of gate pulses on third-quadrant conduction current accumulates over time, which is consistent with the trend of current variation amplitude versus gate pulse width observed in Figure 11a. This behavioral evolution distinctly reveals the transient carrier transport behaviors under pulsed gate biasing: as the negative gate pulse duration extends, the dynamic capture and emission processes of interface traps and the gradual accumulation of holes in the P-well fully develop, continuously modulating the P-well potential and channel conduction capability [Figure 6b], thereby altering the current distribution between the channel and PiN paths.

From a device physics perspective, this pulse-width dependence originates from the transient response of the channel to negative *V*_GS_. When a negative *V*_GS_ is applied, holes accumulate in the P-well and at the oxide interface (as shown in Figure 6a), modifying the surface potential and the *V*th_,eff_. This process is governed by carrier capture and emission at interface traps, introducing a time constant *τ*_trap_. The channel current during the turn-on transient can be modeled as:

(13) $$I_{\mathrm{ch}}(t)=I_{\mathrm{ch},\mathrm{ss}}\left[1-\exp\left(-t/\tau_{\mathrm{trap}}\right)\right]$$

where *I*_ch,ss_ is the steady-state channel current. As the pulse width increases, the channel current approaches *I*_ch,ss_, leading to a higher contribution to the total reverse current, as presented in Figure 6b, where the simulated channel current density increases with longer pulse widths. In addition, the sustained negative *V*_GS_ also alters the P-well potential as described in Section 2. The combination of interface trap filling and P-well potential evolution leads to a progressive increase in channel conduction with pulse width. Consequently, the channel path carries more current, the PiN path less, and the total saturation current rises until a quasi-steady state is reached. Collectively, these results demonstrate that *V*_GS_ is capable of both enabling device reverse conduction and regulating its dynamic response.

Figure 11c illustrates the gate-source voltage waveforms during the turn-on process of the SiC MOSFET under different *V*_GS_. In the simulation, the device is subjected to *V*_GS_ of −5/15 V and 0/15 V, with the SiC material doped with 1.5 × 10^8^ cm^−3^ acceptor defects, the oxide layer containing 1.5 × 10^11^ cm^−3^ acceptor defects, and a SiC/SiO_2_ interface with 1 × 10^10^ cm^−2^ acceptor defects. The results reveal that when the *V*_GS_ is set to −5/15 V, the device exhibits a significantly slower turn-on speed compared to when the *V*_GS_ is set to 0/15 V. This discrepancy is mainly attributed to the enhanced suppression of the MOS channel at negative gate biases, which effectively reduces the ability of the channel to conduct current during the turn-on phase. Consequently, the current flows predominantly through the PiN body diode, which has a longer reverse recovery time and lower conduction efficiency, resulting in a longer turn-on delay. Additionally, the negative *V*_GS_ at −5/15 V leads to an extended Miller plateau time. The longer Miller plateau is linked to the accumulation of holes in the P-well under the negative gate bias (Figure 6), which increases the P-well potential and delays the transition from reverse conduction to full channel conduction. This effect is compounded by the trapping of carriers at the SiC/SiO_2_ interface, which slows down the overall switching dynamics. As a result, negative gate voltages cause slower switching speeds due to a more dominant PiN conduction path and prolonged carrier trapping effects, and the gate voltage of the device directly affects its operating state.

## 4. Conclusions

The parallel conduction behavior between the MOS channel and the PiN body diode in 4H-SiC MOSFETs under third-quadrant operation is systematically investigated. From the perspective of carrier transport, it is found that hole accumulation in the P-well under negative gate bias raises the P-well potential, which is the key reason why partial channel conduction persists even when the gate voltage is negative. The current sharing path and the dynamic transition mechanism between the two conduction paths are shown to be regulated by the gate voltage. This study verifies the significant modulation effect of gate-source bias on both static conduction characteristics and reverse recovery dynamics by combining experimental characterization and TCAD simulation. Furthermore, the influence of negative gate voltage pulses, temperature, and pulse width on reverse conduction behavior is clarified, establishing a physical link between the carrier storage effect and reverse recovery characteristics. These findings deepen the understanding of third-quadrant operation in SiC MOSFETs and provide a theoretical basis for gate-drive design in high-frequency power converters, which is valuable for improving the reliability and performance of SiC power devices in high-power and high-frequency applications.

## Figures and Tables

**Table 1:** Key Parameters of SiC MOSFET Structure.

Structure Parameter	Parameter Values
Cell pitch (μm)	5.2
JFET doping concentration (cm^−3^)	2 × 10^16^
Drift region thickness (μm)	10
Drift region doping (cm^−3^)	8 × 10^15^
Channel length (μm)	0.5
JFET width (μm)	1.2

**Table 2:** Simulated Body Diode Reverse Recovery Values.

*V*_GS_	*Q*_rr_(μC/cm^2^)	*t*_rr_(ns)	*I*_rr_max_(kA/cm^2^)
0 V	484	16.7	198.4
−5 V	648	18.9	209.7

## Data Availability

The original contributions presented in this study are included in the article. Further inquiries can be directed to the corresponding authors.
